# Limited Dispersal and Fragmentation‐Induced Genomic Erosion in Southern Greater Gliders (*Petauroides volans*)

**DOI:** 10.1002/ece3.74204

**Published:** 2026-08-16

**Authors:** Jordyn Clough, Ana Gracanin, Katarina M. Mikac

**Affiliations:** ^1^ Environmental Futures, Faculty of Science, Medicine and Health, School of Science University of Wollongong Wollongong New South Wales Australia; ^2^ Fenner School of Environment and Society Australian National University Canberra Australian Capital Territory Australia

**Keywords:** arboreal marsupial, conservation genetics, genetic erosion, habitat connectivity, kinship, population genomics

## Abstract

Forest fragmentation and isolation are critical threats to arboreal marsupials. The endangered southern greater glider ( *Petauroides volans* ) is vulnerable to the effects of forest loss and fragmentation, yet the species' natural dispersal patterns and genetic outcomes under different habitat contexts remain poorly understood. This study characterises population genomic patterns in southern greater gliders from continuous and isolated forests in southeastern New South Wales, Australia. Using genome‐wide single nucleotide polymorphisms, we genotyped southern greater gliders (*n* = 70) sampled from continuous forests at Mount Lindsey and Tallaganda, and an isolated forest at Seven Mile Beach National Park. Genomic diversity was significantly higher in the continuous forests compared to the isolated forest, which exhibited elevated relatedness and a low effective population size. Fine‐scale spatial genetic structure (0–250 m) was detected across all sites, consistent with limited dispersal. In the continuous forests, inferred parent–offspring pairs were separated by no more than 67 m, suggesting philopatry. In the isolated forest, parent–offspring separation was either no more than 75 m or > 700 m, suggesting both philopatric and dispersing offspring. Co‐denning occurred between related and unrelated conspecifics, including a breeding pair with their adult offspring, indicating sociality beyond maternal and mating associations alone. Our findings indicate that dispersal is typically limited in southern greater gliders and isolated populations are vulnerable to genomic erosion. Conservation strategies must prioritise maintaining forest connectivity to mitigate the genomic consequences of isolation and should consider the species' limited dispersal when planning management actions.

## Graphical abstract

Using genome‐wide SNPs, we characterised population genomic patterns in southern greater gliders from continuous and isolated forests in New South Wales, Australia. Genomic diversity was significantly lower in the isolated population, which exhibited elevated relatedness and a low effective population size, while analyses of fine‐scale spatial genetic structure and parentage revealed typically limited dispersal by southern greater gliders. Our findings highlight the vulnerability of isolated populations to genomic erosion and underscore the need to maintain forest connectivity for the conservation of this endangered marsupial.

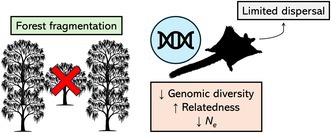

## 1 Introduction

Anthropogenic habitat fragmentation is a threatening process that can disrupt natural ecosystem function and reduce biodiversity (Haddad et al. 2015; Fletcher Jr. et al. 2018; Pinto et al. 2024). Fragmentation can increase extinction risk by isolating populations and eroding genetic diversity through genetic drift and inbreeding (Frankham et al. 2010; Crooks et al. 2017; Ramírez‐Delgado et al. 2022). The dispersal of individuals promotes gene flow and counteracts these genetic effects (Greenwood 1980; Baguette et al. 2013). However, when dispersal is limited, populations develop fine‐scale spatial genetic structure (Peakall et al. 2003; Cutrera et al. 2005; Aguillon et al. 2017). This pattern can arise even in the absence of movement barriers due to intrinsic biological mechanisms (Hazlitt et al. 2006; Quaglietta et al. 2013; Roffler et al. 2014). Understanding genetic structure and dispersal is therefore critical for threatened species conservation, providing insight into species' capacity for natural population recovery following disturbance and the need for management interventions (Moritz et al. 1997; Alves et al. 2023).

Southern greater gliders ( *Petauroides volans* ) are nocturnal gliding possums endemic to the forests of eastern Australia (Kavanagh 2004; van der Ree et al. 2004; McGregor et al. 2020). They are strictly arboreal, requiring trees within gliding distance for movement through the landscape (Kehl and Borsboom 1984; Cunningham et al. 2004) and hollows for denning and shelter (Kehl and Borsboom 1984; Lindenmayer et al. 2004). These dependencies render the species highly vulnerable to habitat loss and fragmentation, with land clearing, logging and fire implicated as key drivers of population declines (Tyndale‐Biscoe and Smith 1969a; Kavanagh and Webb 1998; Lindenmayer et al. 2021; Ashman et al. 2022). This has prompted the species' uplisting to endangered under the Australian federal *Environment Protection and Biodiversity Conservation Act 1999* (Department of Climate Change, Energy, the Environment and Water 2022). These threats, acting alone or in combination, likely exacerbate population isolation by restricting canopy‐dependent dispersal. Despite being a fundamental life‐history trait, dispersal by southern greater gliders remains poorly understood. To date, five dispersal events have been directly recorded or inferred, all from a single study area (Cunningham et al. 2004; Pope et al. 2004; Taylor et al. 2007), while elsewhere one subadult has been observed settling adjacent to its natal range (Henry 1984). Forced displacement has also been recorded following clear‐felling (Tyndale‐Biscoe and Smith 1969a).

Dispersal reflects fitness trade‐offs between the benefits of avoiding inbreeding and kin competition, and the energetic, risk and opportunity costs of movement (Greenwood 1980; Bonte et al. 2012). Southern greater gliders rely on a low‐energy eucalypt leaf diet and have limited body fat reserves, making longer‐distance movement energetically costly (Hume 1999). They exhibit high fidelity to small home ranges (Tyndale‐Biscoe and Smith 1969a; Pope et al. 2004; McGregor et al. 2023; Miritis et al. 2025) and invest only a small proportion of their energy budget in locomotion (Foley et al. 1990). Moreover, greater gliders have steep glide angles and low glide ratios compared to other gliding marsupials (Gracanin et al. 2026), which may constrain their capacity to cross canopy gaps between forest patches. These traits suggest that dispersal distances may be intrinsically limited. Dispersal patterns are also influenced by social organisation and mating systems (Greenwood 1980; Pusey 1987), which in turn are shaped by ecological factors such as resource distribution (Emlen and Oring 1977; Lott 1991; Lawson Handley and Perrin 2007; Martin and Martin 2007). Mating systems can, for example, influence the extent and direction of sex‐biased dispersal (Greenwood 1980). Given that both monogamy and polygyny have been reported in southern greater gliders (Henry 1984; Norton 1988; Pope et al. 2004), resource distribution and the associated mating system may together modulate dispersal. If dispersal is predominantly limited, individuals may not have the capacity to effectively recolonise habitat following disturbances, constraining natural population recovery.

Given that direct observation of dispersal is difficult, genetic approaches provide a powerful means of inferring connectivity and movement (Zenger et al. 2003; Stow et al. 2006). However, genetic studies of southern greater gliders remain limited. Early microsatellite work revealed restricted gene flow within a fragmented pine plantation landscape (Taylor et al. 2002, 2007), but relied on a small number of genetic markers with lower resolution than modern genomic approaches (Morin et al. 2004; Zimmerman et al. 2020). More recent studies of southern greater gliders using genome‐wide single nucleotide polymorphisms (SNPs) have focused on resolving taxonomy (McGregor et al. 2020), broad‐scale genetic structure (Knipler et al. 2023), and genetic decline (Clough et al. 2025). However, fine‐scale genomic patterns and dispersal remain unexplored, especially under different habitat contexts (e.g., isolated vs. continuous forest).

To address this gap, we sampled southern greater glider populations from two large, continuous forests and one small, isolated forest. Using genome‐wide SNPs, we investigated patterns of genomic diversity, population structure and dispersal. We predicted that: (i) the isolated forest population would exhibit lower genomic diversity, higher relatedness and a smaller effective population size compared to the continuous forest populations; (ii) spatial autocorrelation analysis would detect fine‐scale spatial genetic structure across all populations, consistent with limited dispersal; and (iii) parent–offspring pairs (inferred by identity by descent) would predominantly be located in close spatial proximity irrespective of forest isolation.

## 2 Materials and Methods

### 2.1 Glider Sampling and Genotyping

**FIGURE 1:**
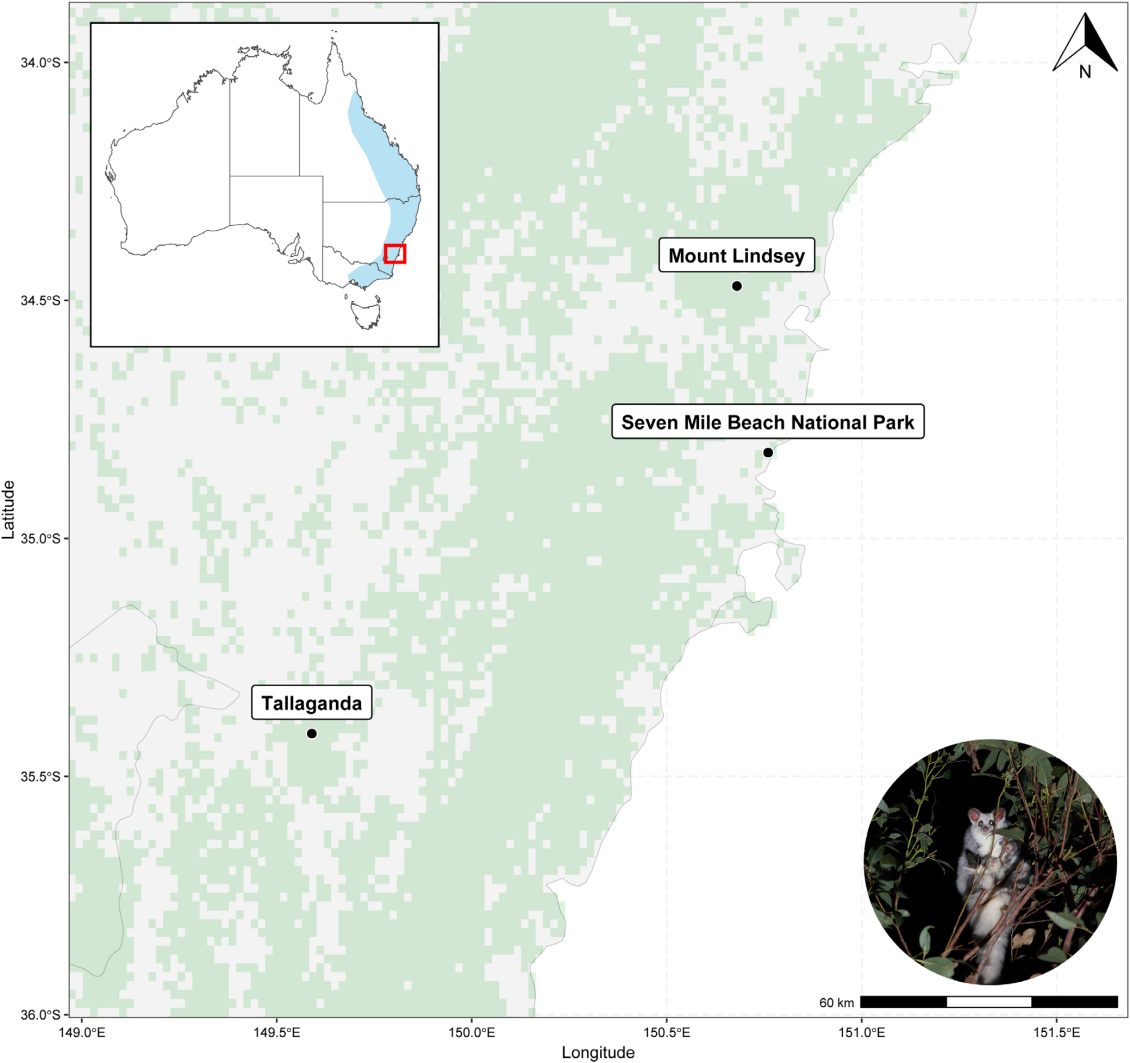
Southern greater glider ( *Petauroides volans* ) sampling locations in southeastern New South Wales, Australia. Native forest cover layer (green) taken from the ‘Forests of Australia (2023)’ spatial dataset (Australian Bureau of Agricultural and Resource Economics and Sciences 2023). Inset shows the distribution of greater gliders (*Petauroides* spp.) across eastern Australia (blue), based on range data from the IUCN Red List of Threatened Species (Johnson and Burbidge 2025). The red box indicates the study area shown in the main panel.

Southern greater gliders were sampled from three eucalypt forests in southeastern New South Wales, Australia (Figure 1; Table A1). The first site, Mount Lindsey (MTL), is situated within a large, continuous landscape of protected native forest (> 90,000 ha). The second site, Tallaganda National Park and State Conservation Area (TAL), comprises two large sections of protected native forest (> 20,000 ha) separated by Tallaganda State Forest, maintaining connectivity to Gourock National Park in the south. The site is located ~150 km southwest of MTL. Notably, the northern portion of TAL, which included private property adjacent to the State Conservation Area that is part of a Biodiversity Conservation Trust, had recently experienced fire and clear‐felling resulting in the reduction of ~50 hollow‐bearing trees (Gracanin et al. 2025). The third site, Seven Mile Beach National Park (SMB), is a small coastal lowland forest (~900 ha) geographically isolated by the clearing of adjacent land for pastoral purposes (c. 1820s) (Murphy 1998) and urban sprawl. It is situated ~40 km southeast of MTL and ~130 km northeast of TAL, lacking connectivity to the nearest contiguous forest located ~10 km west of the Park. Gliders were captured using the branch‐shake method or directly from tree hollows or nest boxes using the climb‐and‐catch method, as previously described (Gracanin et al. 2022). At MTL and SMB, sampling was conducted along linear transects of ~5 km, while at TAL, gliders were sampled from clusters of hollows and nest boxes across a ~20 km extent.

Ear tissue biopsies were collected from adult gliders from MTL (*n* = 9 females, 12 males), TAL (*n* = 19 females, 16 males) and SMB (*n* = 16 females, 4 males) (total *n* = 76). Each individual was sexed, weighed, measured and marked using a unique combination of ear notches created during biopsy to prevent re‐sampling of the same individual. Biopsies were obtained using a 2 mm ear punch (Able Scientific, Australia), which was sprayed with 70% ethanol and flame‐sterilised before use. Tissue was stored in 95% ethanol at −20°C prior to plating.

Tissue samples were sent to Diversity Arrays Technology Pty Ltd. (DArT, Canberra, Australia) for genomic DNA extraction and high‐density single‐nucleotide polymorphism (SNP) genotyping (DArTseq). DNA extraction and SNP genotyping followed protocols optimised for the *Petauroides* genus, as previously described in Knipler et al. (2023). Genotyping was successful for 72 samples, although two were excluded due to high genomic identity consistent with technical duplication. Prior to quality filtering, a total of 28,761 SNPs were represented across the remaining 70 individuals (MTL *n* = 17, TAL *n* = 35, SMB *n* = 18).

### 2.2 Genomic Data Filtering

Filtering of genome‐wide SNPs was performed in R (v. 4.1.1, https://www.r‐project.org) using the dartR package (v. 2.9.9.5) (Gruber et al. 2018; Mijangos et al. 2022). A dataset for population diversity analyses was prepared by filtering as follows: (1) loci with a reproducibility score < 0.95 were removed (*gl.filter.reproducibility*); (2) loci with a read depth < 5 or > 120 were removed (*gl.filter.rdepth*); (3) loci with a call rate < 95% were removed (*gl.filter.callrate*); and (4) monomorphic loci were removed (*gl.filter.monomorphs*). After filtering, a total of 6792 SNPs were retained. To prepare a dataset for population structure and relatedness analyses, the following additional filters were then applied: (5) SNPs with a minor allele frequency (MAF) < 0.01 were removed (*gl.filter.maf*); and (6) secondary SNPs were removed to ensure one SNP per locus (*gl.filter.secondaries*). After subsequent filtering, a total of 5570 SNPs were retained.

### 2.3 Population Genomic Diversity

Genomic diversity analyses were performed on the dataset of 6792 SNPs using dartR. Mean observed heterozygosity (*H*_*o*_), unbiased expected heterozygosity (*uH*_*e*_), and the inbreeding coefficient (*F*_IS_) were calculated using the *gl.report.heterozygosity* function. A permutation‐based randomisation test (*gl.test.heterozygosity*) with 1000 replicates was used to test the significance of pairwise differences in heterozygosity between the populations. Allelic richness (*A*_*r*_) was calculated using the *allelic.richness* function from the hierfstat package (v. 0.5.11) (Goudet 2005). Genomic alpha (*α*_*D*_) and beta (*β*_*D*_) diversity were also calculated using Hill numbers of order *q* = 0 (giving equal weight to all alleles) and *q* = 2 (giving more weight to dominant alleles) with the *gl.report.diversity* function. All diversity metrics were averaged per population.

### 2.4 Effective Population Size

Effective population size (*N*_*e*_) estimates were obtained using the *gl.LDNe* function, calling the NeEstimator program v2 (Do et al. 2014) to calculate *N*_*e*_ using a bias‐corrected version of the linkage disequilibrium method. A ‘random’ mating system was specified and a MAF threshold of 0.05 was applied to reduce bias from rare alleles.

### 2.5 Population Genomic Structure

**FIGURE A1:**
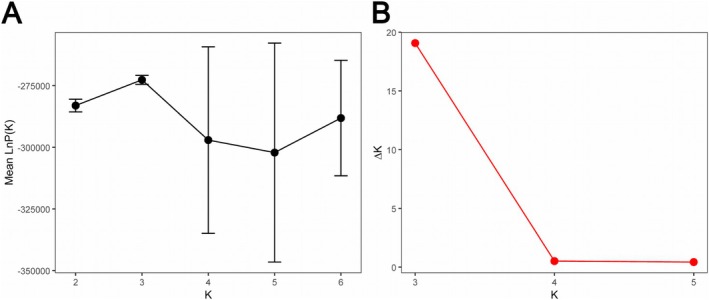
Determination of the optimal number of genetic clusters (*K*) inferred from STRUCTURE analysis. A range of *K* values from *K* = 2 to *K* = 6 were tested. (A) Mean (± SD) log‐likelihood probability (LnP(*K*)) over 20 independent runs for each *K*. (B) Evanno plot showing rate of change in the log probability of data between successive *K* values (Δ*K*). The modal value at *K* = 3, combined with the low variance in LnP(*K*), indicates the most stable population structure.

Population structure analyses were performed on the dataset of 5570 SNPs using dartR. STRUCTURE analysis was run using the *gl.run.structure* function, calling the STRUCTURE program (v. 2.3.4) (Pritchard et al. 2000) with 10,000 burn‐in steps and 50,000 Markov Chain Monte Carlo (MCMC) replicates. Analyses were run for genetic cluster (*K*) values from 1 to 6, with five replicates per *K*. The optimal *K* was determined using the Evanno method (Evanno et al. 2005), as implemented in the *gl.evanno* function (Figure A1). A STRUCTURE bar plot, depicting the most likely number of genetic clusters, was generated using the *gl.plot.structure* function. A Pearson principal components analysis (PCA) was performed as a complementary, model‐free approach to visualise genomic structure using the *gl.pcoa* function, followed by visualisation of the PCA using the *gl.pcoa.plot* function. To quantify genomic divergence among the populations, pairwise *F*_ST_ and corresponding *p* values were calculated with 10,000 bootstraps using the *gl.fst.pop* function.

### 2.6 Spatial Autocorrelation Analysis

To test for fine‐scale spatial genetic structure, spatial autocorrelation was assessed separately for males and females within each population using the *gl.spatial.autoCorr* function from dartR. Following Smouse and Peakall (1999), pairwise genetic autocorrelation coefficients (*r*) were calculated for four distance classes: 0–250 m, 250–500 m, 500–750 m and 750–1000 m. The significance of *r* at each distance class was assessed using 999 permutations to generate a 95% confidence interval (CI) around the null hypothesis of no spatial structure. The 95% CI for the observed *r* value itself was estimated using 999 bootstraps.

### 2.7 Relatedness Estimation and Interpopulation Comparisons

To analyse patterns of relatedness across populations, the common SNP dataset of 5570 SNPs was used. Relatedness was estimated by calculating pairwise coancestry coefficients (*θ*; equivalent to kinship) using the EMIBD9 program (v. 1.1.0.0) (Wang 2022) via the *gl.run*.EMIBD9 function from the dartR.captive package (v. 1.0.2) (Gruber et al. 2018; Mijangos et al. 2022). This method of relatedness estimation is recommended with small sample sizes, specifically for analysing samples from endangered species (Wang 2022). Geographic distances between individuals were calculated from GPS coordinates using the Haversine method implemented in the geosphere package (v. 1.5.20) (Hijmans et al. 2017).

To compare levels of relatedness across different landscape contexts (isolated vs. continuous forest), the mean (± SE) *θ* was calculated for each population. Distributions of *θ* were visualised using violin plots overlaid with boxplots. We tested for significant differences in mean *θ* among populations using the *permTS* function from the perm package (v. 1.0–0.4) (Fay and Shaw 2010), specifying exact permutation tests (‘exact.mc’) with 10,000 Monte Carlo replications. Spatial patterns of relatedness were examined by regressing *θ* against geographic distance. To test whether the relationship between *θ* and distance varied by sex while accounting for population‐level differences, we fitted a linear mixed‐effects model using the lme4 package (v. 1.1.37) (Bates et al. 2015). In the model, *θ* was the response variable, while geographic distance (m), dyad sex composition (female–female vs. male–male), and their interaction were included as fixed effects. Population was included as a random effect to account for baseline differences in *θ* among the sites.

### 2.8 Inference of Parent–Offspring Dyads

Population‐specific SNP datasets were generated to ensure local allele frequencies were accurately represented in estimating intrapopulation relatedness. After filtering to each population, monomorphic loci were removed and only polymorphic loci were retained (MTL = 4687 SNPs, TAL = 4602 SNPs, SMB = 3589 SNPs). To infer PO dyads and distinguish them from other first‐degree relationships, a multivariate classification based on identity by descent (IBD) coefficients was performed.

**FIGURE A2:**
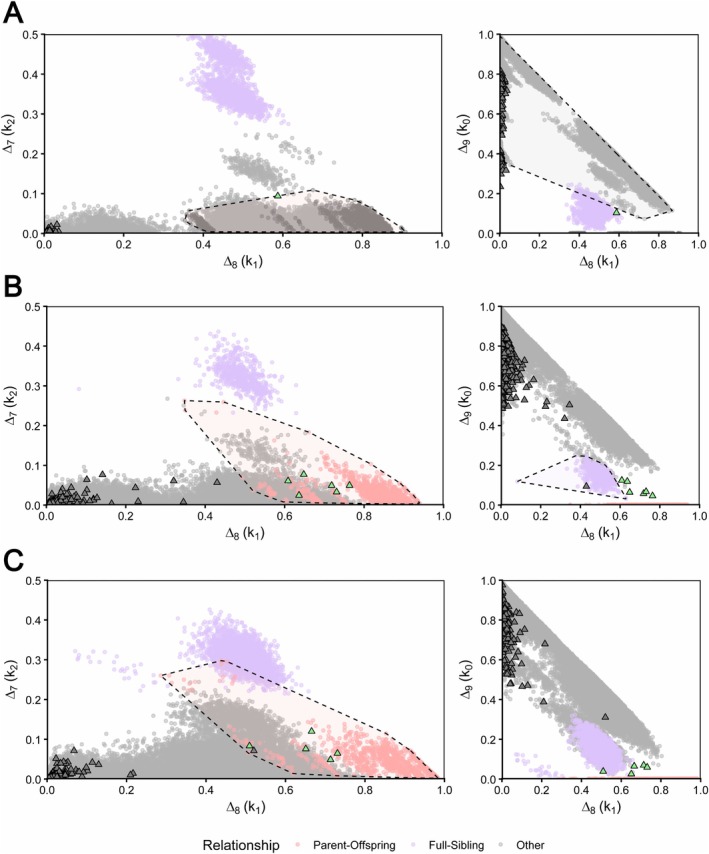
Multivariate genomic classification of empirical parent–offspring dyads at (A) MTL, (B) TAL and (C) SMB. Simulated first‐degree relationships (parent–offspring and full‐sibling) were generated using empirical allele frequencies for each population. Genomic identity by descent coefficients for each dyad were estimated with EMIBD9. Empirical dyads (triangles) were validated as parent–offspring if they fell within the simulated parent–offspring Δ_8_–Δ_7_ distribution (left panels) and were separated from full‐sibling relationships in Δ_8_–Δ_9_ space (right panels). All validated dyads were confirmed to have pairwise coancestry consistent with first‐degree relatives (*θ* ≈ 0.25).

For each population, a simulated dataset was generated using the *gl.sim.offspring* function in R. Mating events (*n* = 250) were simulated using empirical allele frequencies from the population, establishing a pool of known PO (*n* = 1000) and full‐sibling (FS) dyads. IBD coefficients (Δ_1_–Δ_9_) were estimated for all simulated and empirical dyads using the relatedness estimator EMIBD9 (Wang 2022). First‐degree relationships are theoretically distinguished by specific IBD modes: PO share alleles IBD solely via one parent, whereas FS dyads can share alleles via both parents. Therefore, Δ_8_ (probability of sharing one allele IBD) and Δ_7_ (probability of sharing two alleles IBD) were selected as primary discriminators. A classification boundary was defined as the convex hull of the simulated PO distribution in Δ_8_–Δ_7_ space. Empirical dyads falling within the Δ_8_–Δ_7_ boundary were identified as putative PO. These assignments were validated by plotting the dyads in Δ_8_–Δ_9_ space to confirm separation from simulated distributions of non‐first‐degree relationships (Figure A2). Empirical PO were expected to exhibit slightly elevated Δ_9_ values (0–0.1) relative to simulated PO (Δ_9_ = 0), consistent with allelic dropout and low‐level genotyping error introducing apparent Mendelian incompatibilities at a small proportion of loci.

### 2.9 Parent–Offspring Spatial Separation

Natal dispersal patterns were examined by analysing the spatial separation of the inferred PO. The measurement of pairwise geographic distances among kin is a viable proxy for dispersal in wild mammals (Escoda et al. 2017; Hill et al. 2023). Geographic distances between PO were calculated using the Haversine method, as described above. To conservatively delineate dispersal, a threshold was defined based on published home range estimates for southern greater gliders in southeastern Australia (Kavanagh and Wheeler 2004; Pope et al. 2004; McGregor et al. 2023; Miritis et al. 2025). Using the maximum reported home range size (4.88 ha) (Miritis et al. 2025), a circular approximation yielded a radius (*r*) of 125 m. Spatial separation distances of PO ≤ *r* were interpreted as offspring settling within or near the parental home range, while distances > *r* were interpreted as long‐distance movements. Individual developmental stage was assessed using body mass at capture. Individuals were classified following Tyndale‐Biscoe and Smith (1969b): juvenile (< 600 g), immature (601–1000 g) or adult (> 1001 g). Dyads were stratified by sex composition. Spatial separation distances were visualised using strip plots faceted by population.

### 2.10 Resolving Relationships Within Denning Groups

The relationships of gliders captured simultaneously from the same hollow or nest box (only at TAL) were inferred to determine the composition of denning groups. To account for stochastic variation around theoretical expectations for *θ* (Wright 1922), the simulated TAL dataset was used for reference distributions of *θ*. Classification thresholds were defined as the midpoint between the lower 95% CI of the higher degree of relationship and the upper 95% CI of the lower degree of relationship.

Dyads with *θ* below the upper 95% CI of the unrelated distribution (*θ* < 0.065) were classified as unrelated (indistinguishable from background population relatedness). Relationships were assigned as: first‐degree (*θ* ≥ 0.236); second‐degree (0.097 ≤ *θ* < 0.236); distant (0.065 ≤ *θ* < 0.097); or unrelated (*θ* < 0.065). To resolve ambiguous relationships where empirical values conflicted with standard Mendelian expectations, inbreeding was also assessed using the individual inbreeding coefficient (*F*) and genomic IBD coefficients Δ_1_…Δ_6_ estimated by EMIBD9 (Wang 2022). The Δ_1_…Δ_6_ coefficients effectively distinguish inbred from outbred relationships by detecting excess genomic IBD characteristic of consanguinity.

## 3 Results

### 3.1 Population Genomic Diversity

Genomic diversity was higher in the continuous forest populations (MTL, TAL) compared to the isolated forest population (SMB) (Table 1). Expected heterozygosity was highest at MTL (mean 0.212 ± 0.002 SE), intermediate at TAL (0.186 ± 0.002), and lowest at SMB (0.157 ± 0.002), with all pairwise comparisons being statistically significant (*p* < 0.001; Table 1).

**TABLE 1:** Genomic diversity in southern greater glider populations at Mount Lindsey (MTL), Tallaganda (TAL) and Seven Mile Beach National Park (SMB).

Population	*H*_*o*_	_*u*_ *H*_*e*_	*F*_IS_	Ar	^*0*^ *D*_*α*_	^*2*^ *D*_*α*_
MTL	0.172 (± 0.002)	0.212 (± 0.002)	0.173 (± 0.004)	1.75 (± 0.005)	1.77	1.33
TAL	0.164 (± 0.002)	0.186 (± 0.002)	0.115 (± 0.003)	1.65 (± 0.005)	1.76	1.30
SMB	0.145 (± 0.002)	0.157 (± 0.002)	0.075 (± 0.003)	1.55 (± 0.006)	1.57	1.25

*Note:* Analysis based on 6792 SNPs. Diversity metrics include observed heterozygosity (*H*_*o*_), unbiased expected heterozygosity (_*u*_ *H*_*e*_), inbreeding coefficient (*F*_IS_), allelic richness (*Ar*) and genomic alpha diversity indices (^*0*^ *D*_*α*_, ^*2*^ *D*_*α*_). Values show mean (± SE).

Mean allelic richness was highest at MTL (1.75 ± 0.005), followed by TAL (1.65 ± 0.005) and SMB (1.55 ± 0.006) (Table 1). MTL and TAL had higher alpha diversity than SMB, both in terms of rare alleles (^*0*^ *D*_*α*_) and dominant alleles (^*2*^ *D*_*α*_), although these values were low overall (Table 1). Beta diversity was higher for rare alleles (^*0*^ *D*_*β*_ = 1.17–1.20) than for dominant alleles (^*2*^ *D*_*β*_ = 1.02–1.07), indicating the populations differed more in rare than dominant alleles (Table A2).

### 3.2 Effective Population Size

Effective population size estimates were highest at MTL (*N*_*e*_ = 139.4 [95% CI: 85.4, ∞]), followed by TAL (*N*_*e*_ = 89.6 [95% CI: 57.9, 199.9]), then SMB (*N*_*e*_ = 34.9 [95% CI: 20.9, 84.4]).

### 3.3 Population Genomic Structure

**FIGURE 2:**
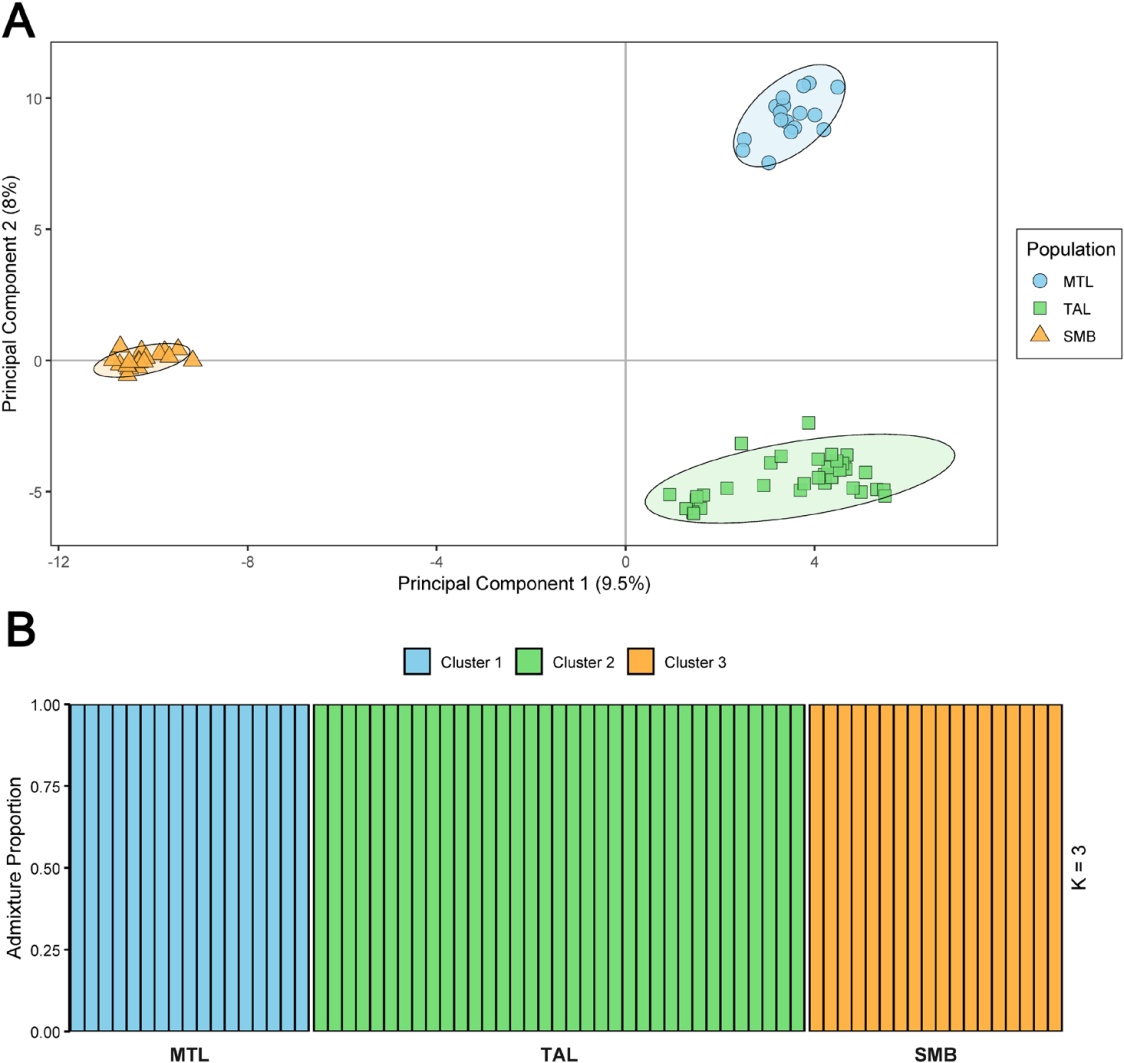
Genomic structure of southern greater gliders from Mount Lindsey (MTL), Tallaganda (TAL) and Seven Mile Beach National Park (SMB). (A) PCA plot visualising population genomic structure. (B) STRUCTURE plot identifying genetic clusters among gliders (*K* = 3). Analysis based on 5570 SNPs.

Principal components analysis of SNP genotypes revealed clear separation among the three populations (Figure 2A). Individuals from each population formed distinct, non‐overlapping clusters, while samples from SMB clustered more tightly than those from MTL and TAL. Principal components 1 and 2 explained 9.5% and 8.0% of the total genomic variation, respectively (Figure 2A).

Genomic differentiation was significant among the three populations (*p* < 0.001). The highest degree of divergence was detected between SMB and MTL (*F*_ST_ = 0.169), followed by SMB and TAL (*F*_ST_ = 0.139), while the lowest divergence was detected between MTL and TAL (*F*_ST_ = 0.116).

**FIGURE A3:**
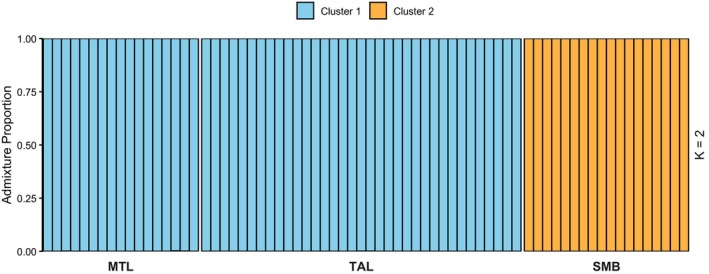
STRUCTURE plot (*K* = 2) identifying genetic clusters among southern greater gliders from Mount Lindsey (MTL), Tallaganda (TAL) and Seven Mile Beach National Park (SMB).

Population structure modelling via STRUCTURE indicated *K* = 3 as the most likely number of genomic clusters (Figure 2B). At *K* = 3, MTL, TAL and SMB individuals were assigned to distinct clusters (blue, green and orange, respectively) with high assignment probabilities (*Q* > 0.99). No evidence of recent admixture among the populations was detected (Figure 2B). At *K* = 2, all SMB individuals were assigned to one cluster, while MTL and TAL individuals were grouped together in a separate cluster, indicating genomic separation of SMB from the continuous forest populations (Figure A3).

### 3.4 Spatial Genetic Structure

**FIGURE 3:**
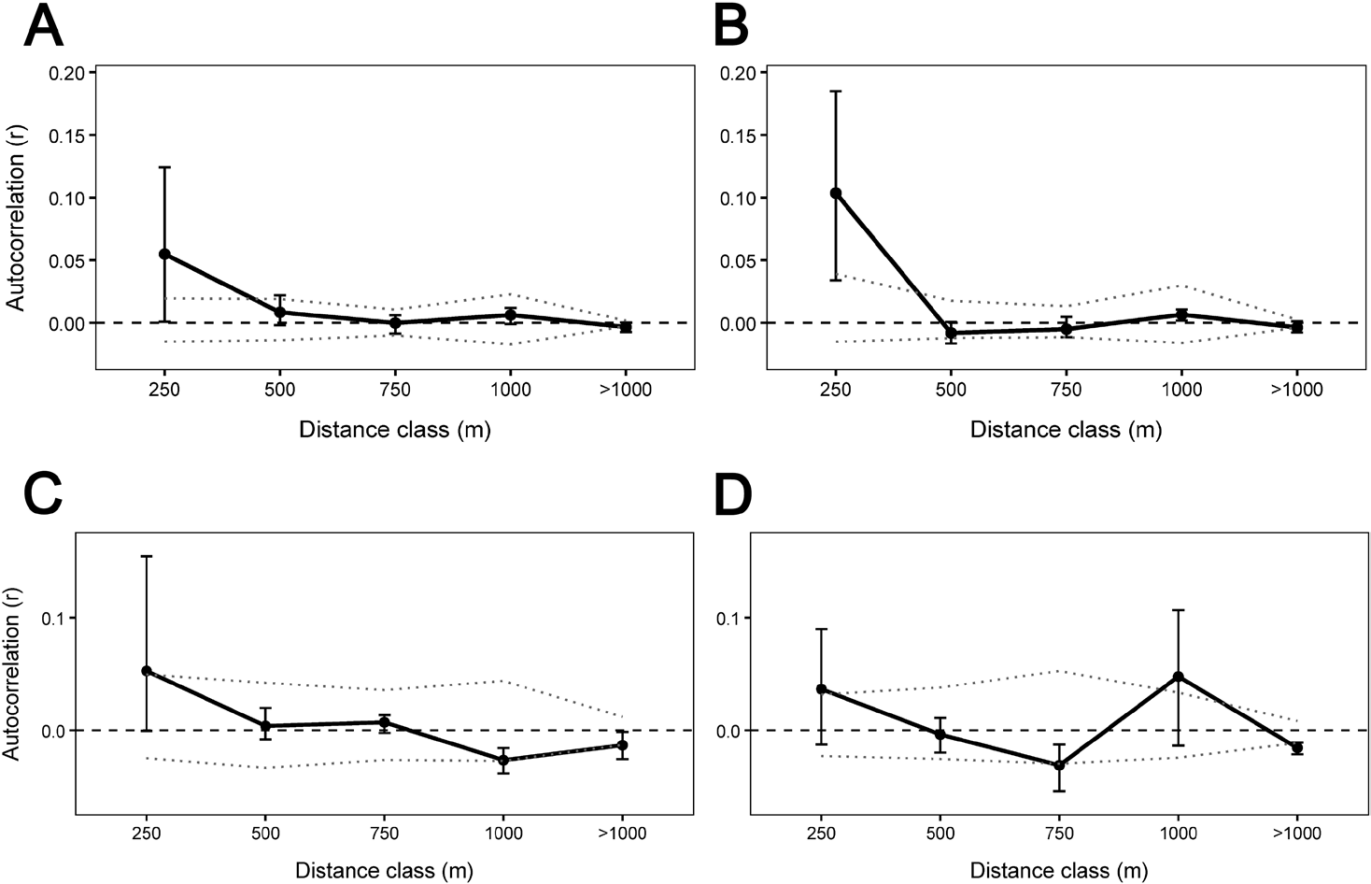
Spatial autocorrelation correlograms showing the relationship between genetic and geographic distances for southern greater gliders: (A) females from Tallaganda, (B) males from Tallaganda, (C) males from Mount Lindsey, and (D) females from Seven Mile Beach National Park. The solid line represents the observed autocorrelation (*r*), with error bars indicating the 95% confidence interval at each distance class based on bootstrap tests (9999 bootstraps). The dotted lines represent the 95% upper and lower confidence limits around random expectations. The > 1000 m distance class includes all remaining pairwise comparisons up to the maximum sampling extent (~20 km for A, B; ~5 km for C, D).

Fine‐scale spatial genetic structure was detected for both sexes based on spatial autocorrelation analysis (Figure 3). At TAL, the only site with sufficient data for a direct comparison between the sexes, significant positive spatial autocorrelation was detected at 0–250 m for both TAL females (*r* = 0.055, *p* < 0.01) and TAL males (*r* = 0.104, *p* < 0.01) (Figure 3A,B). This fine‐scale structuring was consistent across different site contexts, with significant positive spatial autocorrelation also detected at 0–250 m for MTL males (*r* = 0.053, *p* = 0.01) (Figure 3C) and SMB females (*r* = 0.037, *p* < 0.01) (Figure 3D). Analysis could not be performed on MTL females and SMB males due to insufficient dyads captured in short‐range distance classes.

Significant negative spatial autocorrelation was detected in the > 1000 m distance class for all tested site–sex combinations (Figure 3). Additionally, MTL males exhibited significant negative spatial autocorrelation at 750–1000 m (*r* = −0.027, *p* = 0.03) (Figure 3C), while SMB females showed significant spatial autocorrelation at multiple intermediate distance classes, with negative autocorrelation at 500–750 m (*r* = −0.031, *p* = 0.01) and positive autocorrelation at 750–1000 m (*r* = 0.048, *p* < 0.01) (Figure 3D).

### 3.5 Patterns of Relatedness

**FIGURE 4:**
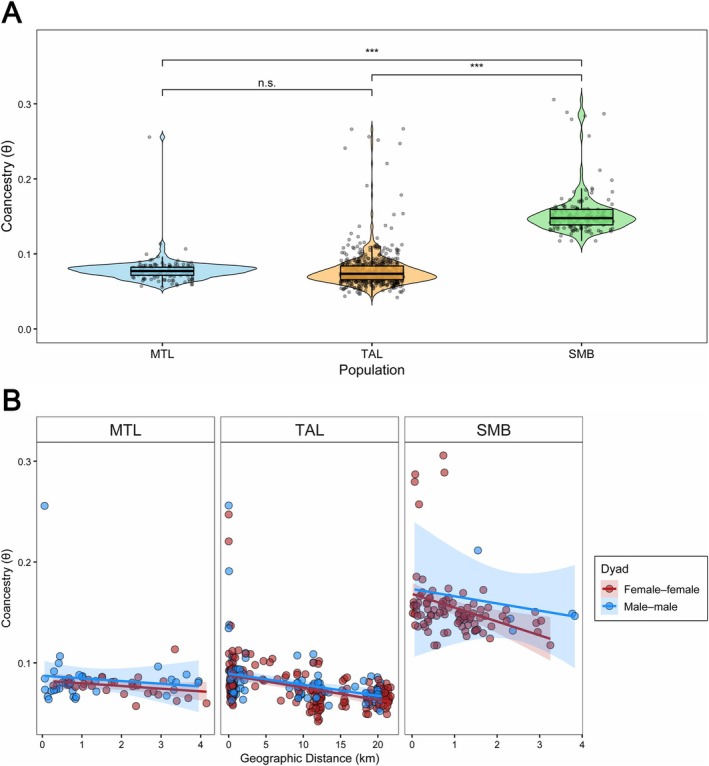
Patterns of pairwise coancestry (*θ*) within southern greater glider populations at Mount Lindsey (MTL), Tallaganda (TAL) and Seven Mile Beach National Park (SMB). (A) Violin plots display the probability density of *θ* values and internal boxplots indicate the median and interquartile range (whiskers extend to 1.5 × interquartile range). Points represent dyads. *** = *p* < 0.001; n.s. = *p* > 0.05. (B) Relationship between pairwise coancestry and geographic distance. Regression lines are shown for female–female (red) and male–male (blue) dyads. Shading represents the 95% confidence interval around the regression line. Note the differing x‐axis scales reflecting the maximum sampling extent at each site.

Relatedness within each population varied by landscape context (Figure 4A). Mean pairwise coancestry was significantly higher in the isolated population (SMB: *θ* = 0.154 ± 0.003 SE) compared to the continuous forest populations (MTL: *θ* = 0.078 ± 0.002 SE; TAL: *θ* = 0.078 ± 0.001 SE) (both *p* < 0.001). However, mean pairwise coancestry did not differ significantly between the two continuous forest populations (*p* = 0.99).

Pairwise coancestry declined with geographic distance within all populations, consistent with a general pattern of isolation by distance (Figure 4B). To formally test for sex differences while controlling for population‐level variation, we fitted a linear mixed‐effect model. While the model confirmed a significant decline of coancestry with distance overall (*β* = −1.27e^−06^, *p* < 0.001), it did not detect a significant interaction between sex and distance (*p* = 0.76), indicating comparable declines between the sexes.

### 3.6 Parent–Offspring Spatial Separation

**FIGURE 5:**
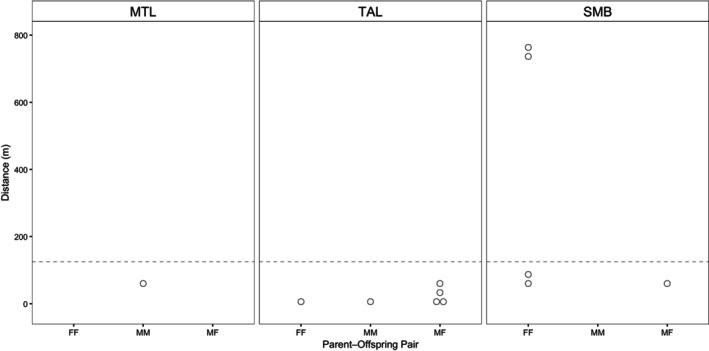
Spatial separation of parent–offspring pairs at Mount Lindsey (MTL), Tallaganda (TAL) and Seven Mile Beach National Park (SMB). Pairs are grouped by sex composition (FF = female–female; MM = male–male; MF = male–female). The dashed line represents a home range radius of 125 m (corresponding to a 4.88 ha home range), based on the maximum reported home range size for southern greater gliders in southeastern Australia (Miritis et al. 2025). Data points at 0 m represent pairs captured from the same den.

Parent–offspring pairs were separated by distances ranging from 0 to 761 m, with 10 of 12 pairs found at short distances (0–75 m) (Figure 5; Table 2). In the continuous forests (MTL and TAL), all inferred PO pairs were short‐distance (0–67 m, *n* = 7), while the isolated forest (SMB) contained both short‐distance (51–75 m, *n* = 3) and long‐distance (737–761 m, *n* = 2) pairs.

**TABLE 2:** Inferred parent–offspring pairs at Mount Lindsey (MTL), Tallaganda (TAL) and Seven Mile Beach National Park (SMB).

Population	Individuals	Sex	Body mass (g)	*θ*	Distance (m)
MTL	#44	M	1285	0.267	58
	#71	M	1070		
TAL	#05	M	1235	0.250	37
	#08	F	1442		
	#06	M	1002	0.252	0
	#50	M	1195		
	#06	M	1002	0.245	0
	#08	F	1442		
	#11	M	1015	0.282	0
	#14	F	1013		
	#40	M	1100	0.267	67
	#04	F	—		
	#41	F	995	0.248	0
	#30	F	1320		
SMB	#80	M	1235	0.256	51
	#77	*F*	900		
	#17	*F*	1585	0.285	63
	#20	*F*	665		
	#04	*F*	1545	0.295	*737*
	#60	*F*	905		
	#09	*F*	1590	0.286	*761*
	#77	*F*	900		
	#10	*F*	1515	0.270	*75*
	#100	*F*	690		

*Note:* Sex, body mass (g), pairwise coancestry (*θ*) and the spatial separation distance of each pair in metres (m) are indicated. A distance of 0 m indicates the pair were captured simultaneously from the same den.

Abbreviations: F, female; M, male.

**FIGURE 6:**
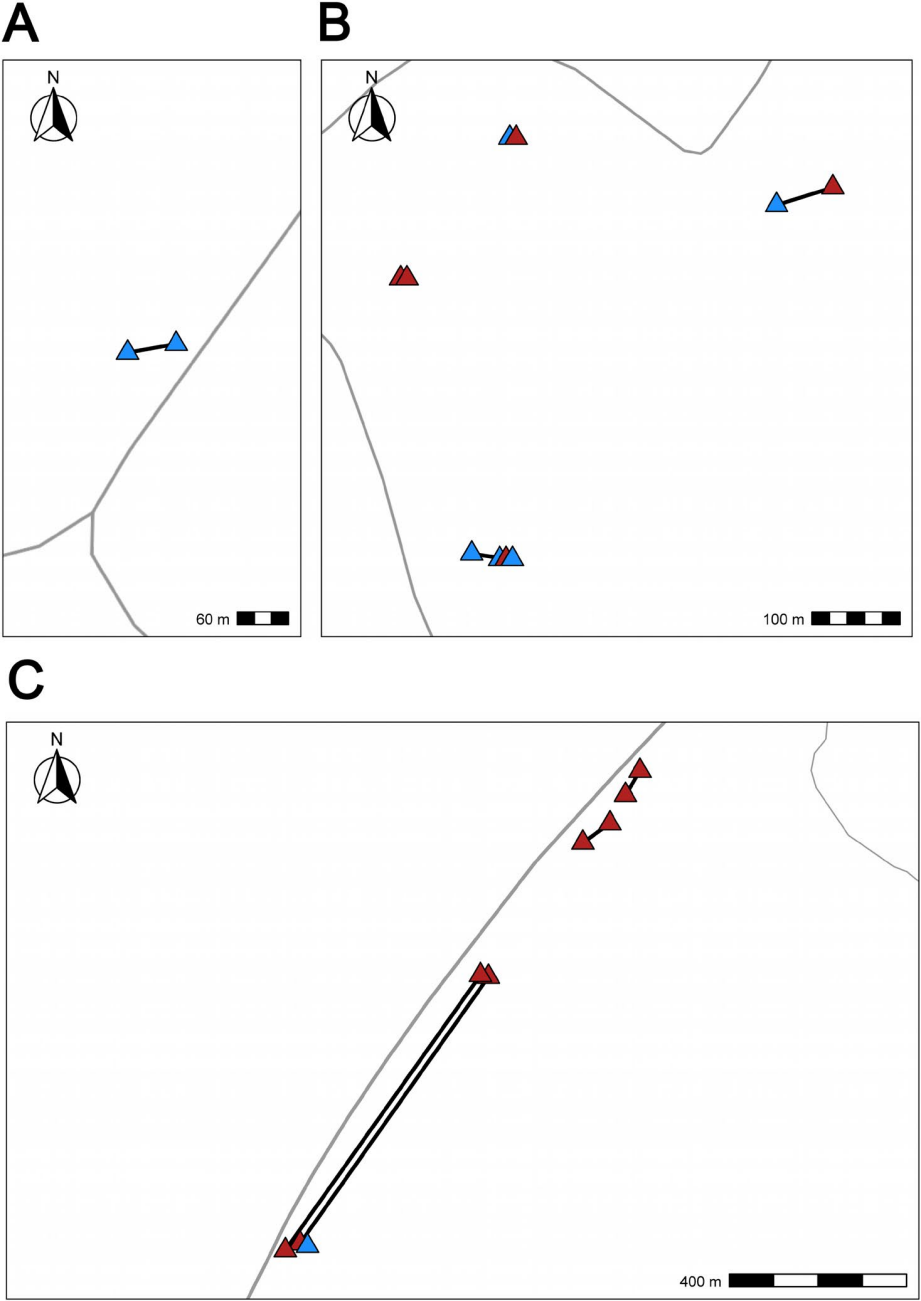
Spatial distribution of parent–offspring pairs at (A) Mount Lindsey, (B) Tallaganda and (C) Seven Mile Beach National Park. Triangles represent individuals coloured by sex (red = female; blue = male). Solid black lines indicate inferred parent–offspring relationships. Symbols for pairs captured from the same den (TAL only) are displayed with a slight horizontal offset for visibility.

At MTL, one father–son relationship was inferred (both adults > 1001 g), with the pair separated by 58 m (Figure 6A). At TAL, six PO relationships were inferred, comprising three independent pairs and one mother–father–son triad (Figure 6B). Of the independent pairs, one mother–daughter pair and one male–female pair were co‐denning (0 m), while another male–female pair was separated by 67 m. The mother–father–son triad was co‐denning (0 m), with the female from this triad sharing another PO relationship with a male located 37 m away. Four of the five TAL offspring were adults (> 1001 g).

At SMB, five PO relationships were inferred, comprising three independent mother–daughter pairs and one mother–father–daughter triad (Figure 6C). Short‐distance pairs (51–75 m) included two independent mother–daughter pairs and the father–daughter pair from the triad. Long‐distance pairs (737–761 m) included one independent mother–daughter pair and the mother–daughter pair from the triad. All four SMB offspring were immature (665–970 g).

### 3.7 Composition of Denning Groups

Denning groups comprised both related and unrelated individuals (Table 3). Eight denning groups were pairs, with male–female pairs the most common (*n* = 6). Three were unrelated, while the other three ranged from distant to first‐degree relationships. Both female–female pairs (*n* = 2) were first‐degree relatives, including one mother–daughter pair and one pair of full‐siblings. No male–male pairs were observed. Two denning groups were triads. The first consisted of a breeding pair (unrelated to each other) with their male offspring. The second consisted of two distantly related males and a female unrelated to either male.

**TABLE 3:** Composition of southern greater glider denning groups.

Denning group	Den type	Dyad	Sex	*θ*	Degree of relationship	Inferred relationship
Triad	NB	#50–#06	MM	0.252	1st	Father–son
		#08–#06	MF	0.245	1st	Mother–son
		#50–#08	MF	0.006	Unrelated	Breeding pair
Triad	H	#02T–#20T	MM	0.090	< 2nd	Distant relative
		#02T–#70T	MF	0.058	Unrelated	—
		#20T–#70T	MF	0.050	Unrelated	—
Pair	NB	#41–#30	FF	0.247	1st	Mother–daughter
Pair	NB	#110–#80	FF	0.248	1st	Full‐siblings^a^
Pair	NB	#11–#14	MF	0.281	1st	Parent–offspring
Pair	NB	#04B–#07	MF	0.108	2nd	Close relative^b^
Pair	NB	#01T–#03T	MF	0.075	< 2nd	Distant relative
Pair	H	#21–#20	MF	0.022	Unrelated	—
Pair	NB	#01–#04	MF	0.062	Unrelated	—
Pair	NB	#25T–#23T	MF	0.050	Unrelated	—

*Note:* Data represent all instances where ≥ 2 gliders were captured simultaneously from the same hollow (H) or nest box (NB). Degree of relationship indicates the genomic classification based on simulated pairwise coancestry (*θ*) thresholds at Tallaganda (first‐degree: *θ* ≥ 0.236; second‐degree: 0.097 ≤ *θ* < 0.236; distant relative: 0.065 ≤ *θ* < 0.097; unrelated: *θ* < 0.065).

Abbreviations: F, female; M, male.

^a^ Inferred to be an inbred full‐sibling relationship based on genomic identity by descent sharing. See Appendix S1 for details.

^b^ Inference based on *θ* consistent with second‐degree relationship (e.g., half‐siblings, avuncular or grandparent–grandoffspring).

## 4 Discussion

This study provides new insights into southern greater glider dispersal, sociality and the genomic consequences of forest fragmentation and isolation. In agreement with our predictions: (i) the isolated population exhibited reduced genomic diversity, elevated relatedness and a lower effective population size (*N*_*e*_) compared to the populations in continuous forests; (ii) fine‐scale spatial genetic structure (0–250 m) was detected across all sites, consistent with limited dispersal; and (iii) offspring were predominantly in close proximity to parents, with 10 of 12 PO pairs separated by < 75 m. Our findings demonstrate that dispersal is typically limited in southern greater gliders and isolated populations are vulnerable to genomic erosion, underscoring the critical importance of maintaining forest connectivity to support population genetic health.

### 4.1 Genomic Consequences of Forest Fragmentation and Isolation

Our findings demonstrate how forest connectivity shapes the genomic resilience of southern greater glider populations. This is evident in the contrast between populations occupying large, continuous forests (MTL and TAL), which retain high genomic diversity and larger *N*_*e*_, and the geographically isolated SMB population. The SMB population exhibits characteristics of a closed population, including low genomic diversity, high relatedness and a critically low *N*_*e*_, which together signal a high risk of local extinction (Frankham et al. 2010). Knipler et al. (2023) previously reported comparable patterns at isolated locations across the species' southern range in NSW. Isolated and restricted to a small number of individuals (Vinson et al. 2021; Mulley et al. 2024), the SMB population is vulnerable to an ‘extinction vortex’, a positive feedback loop where inbreeding depression reduces average fitness, driving the population size even lower (Caughley 1994). This trajectory has been independently supported by population viability analysis (Mulley et al. 2024) and evidence of temporal genetic decline (Clough et al. 2025). The refined SMB *N*_*e*_ point estimate (34.9) remains well below the recommended management thresholds of *N*_*e*_ > 100 to avoid inbreeding depression in the short term and *N*_*e*_ > 1000 to maintain long‐term evolutionary potential (Frankham et al. 2014). While the continuous forest populations exhibited substantially larger *N*_*e*_ than SMB, they still fall well below the long‐term threshold, with the TAL point estimate (*N*_*e*_ = 89.6) sitting just below the short‐term threshold. Given the endangered status of the southern greater glider (Department of Climate Change, Energy, the Environment and Water 2022), this highlights that even populations in continuous forest can operate at relatively low *N*_*e*_ and therefore warrant ongoing monitoring and conservation attention.

Both PCA and STRUCTURE identified three distinct genetic clusters in our study, confirming that restricted gene flow is driving population divergence. Given the significant genomic differentiation observed among the clusters, the studied populations should be recognised as separate management units (Funk et al. 2012) to ensure their local genomic diversity is preserved. However, despite substantial geographic separation (~150 km) between MTL and TAL, their pairwise differentiation (*F*_ST_ = 0.116) and hierarchical clustering reveal they retain significantly more genomic similarity to one another than either does to SMB (*F*_ST_ = 0.139–0.169). This suggests that geographic isolation by the cleared agricultural matrix surrounding the Park, rather than distance alone, is the dominant driver of the genomic divergence observed at SMB. Comparable genetic differentiation driven by agricultural matrix isolation has been documented in the common ringtail possum ( *Pseudocheirus peregrinus* ) (Lancaster et al. 2016). The depth of divergence at SMB likely reflects the cumulative effects of historical population bottlenecks following European settlement, land clearing and isolation of the Park (c. 1820s) (Murphy 1998). Given an estimated generation time of 7–8 years (Woinarski et al. 2014), the SMB population has likely been genetically isolated for ~25–29 generations. This timeframe is ample for substantial effects of genetic drift to accumulate, particularly given the critically low effective population size and the expected rate of heterozygosity loss (~1/(2*N*_*e*_) per generation) (Frankham et al. 2010).

Without forest connectivity to facilitate dispersal and gene flow, forest‐dependent mammals experience drift‐induced population subdivision and a loss of genetic diversity (Mech and Hallett 2001; Frankham et al. 2010; Sharma et al. 2013; Christie and Knowles 2015). Pope et al. (2004) proposed that dispersal in southern greater gliders may be restricted where the surrounding matrix provides few feeding or denning opportunities. Consistent with this, Taylor et al. (2007) showed that while dispersal through pine plantation matrix was possible, it was sufficiently restricted to maintain genetic differentiation among eucalypt patches. The isolation of SMB represents the extreme endpoint of fragmentation. Unlike pine plantation matrix, which can act as a permeable barrier that allows for some southern greater glider dispersal (Taylor et al. 2007), the cleared agricultural matrix functions as a hard barrier due to the species' strict arboreal lifestyle (Tyndale‐Biscoe and Smith 1969a; Kehl and Borsboom 1984; Gracanin and Mikac 2023). Consequently, distinct patterns of genomic diversity and relatedness have emerged. In continuous forests, immigration introduces new genetic material, keeping relatedness low and genomic diversity high. In isolated forests like SMB, dispersal cannot occur across cleared agricultural matrix and alleles become fixed or lost within a finite gene pool. In the absence of immigration, genetic drift and inbreeding are expected to elevate relatedness and decrease genetic diversity (Frankham et al. 2010). These predictions are consistent with the significantly reduced genomic diversity and nearly two‐fold higher relatedness at SMB (mean *θ* = 0.154) compared to MTL and TAL (mean *θ* = 0.078). Ultimately, the erosion of genomic diversity and accumulation of relatedness observed at SMB illustrates the broader genomic trajectory expected for populations isolated by forest fragmentation.

### 4.2 Limited Dispersal and Fine‐Scale Genetic Structure

Our findings provide evidence that dispersal is typically limited in southern greater gliders. In the continuous forests (MTL and TAL), we detected fine‐scale genetic structure (0–250 m) in the form of significant positive spatial autocorrelation for both males and females. This fine‐scale positive autocorrelation delineates the kin neighbourhood: the spatial scale over which restricted dispersal leads to an aggregation of related individuals who share higher genomic similarity than expected by chance (Banks and Peakall 2012). Similar patterns have been documented in other Australian possums characterised by limited dispersal and high philopatry (Stow et al. 2006; Yokochi et al. 2016). As observed in the common brushtail possum ( *Trichosurus vulpecula* ), fine‐scale structuring can persist in both sexes when overall dispersal distances are limited, even if one sex disperses further (Stow et al. 2006). These population‐level signatures are therefore consistent with limited dispersal and gene flow beyond the kin neighbourhood. Taylor et al. (2007) found dispersal of southern greater gliders between fragmented eucalypt patches, through an intervening pine matrix, to be infrequent and difficult to detect, with most identified dispersers originating from unsampled or unresolved sources. Our findings extend this by demonstrating that dispersal, and subsequent gene flow, is typically limited even in the absence of a matrix barrier. This suggests that limited dispersal reflects the species' conservative ranging behaviour, rather than strictly the impermeability of a surrounding matrix.

This baseline of limited dispersal is corroborated by the spatial distribution of PO pairs in the continuous forests. Five of six offspring were adults (> 1001 g) (Tyndale‐Biscoe and Smith 1969b) and all were located within a single home range radius of their parents (0–67 m), indicating that offspring reaching adulthood tend to persist in close proximity to their parents' ranges (i.e., remain philopatric). Two of these relationships were father–son, confirming that philopatric offspring can be male, one of which involved an adult male co‐denning with both parents. A third comprised an adult mother and an immature daughter on the cusp of adulthood (995 g) (Tyndale‐Biscoe and Smith 1969b), consistent with philopatry in females. The presence of philopatric males deviates from the typical mammalian expectation of male‐biased dispersal (Greenwood 1980; Pusey 1987), although dispersal patterns vary considerably across mammals (Mabry et al. 2013). Limited dispersal is likely reinforced by physiological constraints, as greater gliders rely on a low‐energy folivorous diet and have limited body fat reserves, making dispersal energetically costly (Hume 1999). Our empirical findings in the continuous forests support earlier ecological observations of conservative ranging behaviour and high site fidelity in the species (Tyndale‐Biscoe and Smith 1969a; Henry 1984; Kehl and Borsboom 1984; Kavanagh and Wheeler 2004; Pope et al. 2004).

### 4.3 Dispersal and Gene Flow in an Isolated Forest

Against this baseline, dispersal and gene flow patterns in the isolated forest population differed from those observed in the continuous forests. Divergent spatial patterns were evident among sampled females at SMB. While SMB females retained significant positive autocorrelation at the fine scale (0–250 m), consistent with the continuous forest kin neighbourhoods, they also exhibited significant negative autocorrelation at 500–750 m followed by significant positive autocorrelation at 750–1000 m. This pattern suggests heterogeneity in female movement, where some related females remain philopatric while others disperse beyond the kin neighbourhood. While the continuous capture of individuals along a 5 km linear transect at SMB suggests this re‐emergence of positive autocorrelation reflects true dispersal rather than an artefact of patchy sampling, we acknowledge that other unmeasured factors could theoretically contribute to such spatial genetic patterns. This population‐level signature is further substantiated by mother–daughter pairs at SMB being separated by either < 75 m or > 700 m, but not at intermediate distances. Given the limited number of pairs, this pattern should be interpreted with caution. However, its agreement with the independent spatial autocorrelation result lends support to an inference of female dispersal at SMB.

Unlike the continuous forests, all four offspring at SMB were immature (665–970 g) (Tyndale‐Biscoe and Smith 1969b). Given that the home ranges of southern greater gliders are typically small (1–4 ha) (Kavanagh and Wheeler 2004; Pope et al. 2004; McGregor et al. 2023; Miritis et al. 2025), > 700 m separation between mother–daughter pairs far exceeds the scale of daily movements and is consistent with natal dispersal, although one of these daughters was also recorded within 75 m of her father. Dispersal events are rarely documented in the species. While Tyndale‐Biscoe and Smith (1969a) used mark‐recapture to record displaced gliders relocating up to 2.8 km immediately following clear‐felling of eucalypt forest, these movements were involuntary responses to habitat destruction rather than voluntary dispersal; nonetheless, they demonstrate the species' capacity for movement over such distances. Henry (1984) observed a subadult taking up residence in unoccupied forest adjacent to its natal range, although the distance was not measured. In a fragmented pine plantation, Pope et al. (2004) and Cunningham et al. (2004) used radiotracking to record a single dispersal event of ~1 km between remnant eucalypt patches, while Taylor et al. (2007) used parentage analysis to infer four additional inter‐patch dispersers, including a subadult female that moved ~7 km. Our findings build upon these sparse records, documenting immature female gliders located > 700 m from their mothers, suggestive of dispersal. Whether immature offspring in close proximity to parents remain nearby through adulthood or eventually disperse, in a form of delayed natal dispersal (Efford 1998; Sparkman et al. 2010; Mayer et al. 2017), remains an open question.

We offer two possible explanations for this pattern in the isolated forest. First, it may represent the upper tail of an otherwise limited dispersal distribution, as rare, long‐distance dispersal events have been recorded in the species (Taylor et al. 2007). Under this explanation, the pattern may have been detected at SMB but not elsewhere owing to chance or to differences in the proportion of each population sampled. Second, it may reflect a context‐dependent shift in dispersal behaviour under isolation. Ecological theory predicts that social organisation and mating systems, which influence dispersal patterns, can vary among populations in response to factors such as resource distribution (Emlen and Oring 1977; Greenwood 1980). Such resource‐driven variation has been empirically demonstrated in the mountain brushtail possum ( *Trichosurus cunninghami* ), another hollow‐dependent arboreal marsupial, whereby female spacing and the mating system shift in response to the availability of den and feed trees (Martin and Martin 2007). Variable social systems previously reported in southern greater gliders (Henry 1984; Pope et al. 2004) may reflect a similar dynamic, providing reason to expect that dispersal patterns vary similarly. At SMB specifically, female dispersal may be driven by intensified competition for limited resources (Greenwood 1980; Martin and Martin 2007) or the need for inbreeding avoidance (Pusey and Wolf 1996), though the exact drivers remain unclear. While our data cannot disentangle these two explanations, the second is consistent with the ecological theory outlined above. In either case, although dispersal in southern greater gliders is typically limited, it was detected in the isolated population. Together with the genomic signatures described, these findings extend evidence that natural population dynamics of arboreal marsupials are affected by forest fragmentation and isolation (Laurance 1990; Lindenmayer and Lacy 1995; Taylor et al. 2007; Lancaster et al. 2011, 2016; Youngentob et al. 2013; Knipler et al. 2022).

### 4.4 Sociality in Den Sharing

Southern greater gliders exhibit a social tolerance in their den sharing behaviour, co‐denning with both related and unrelated conspecifics. This pattern is more nuanced than some prior descriptions of the species as solitary suggest (Cunningham et al. 2004; Eyre 2006). While denning associations have been described previously, the relatedness of the individuals has generally been inferred rather than measured. Henry (1984) reported den sharing between breeding pairs, with some males denning with more than one female. Lindenmayer et al. (2004) subsequently reported sharing of den trees between parents and offspring of the same sex, and between unrelated pairs of the opposite sex. In both studies, these relationships rested on observed breeding and parental behaviours, with individuals otherwise presumed unrelated. We extend this work by pairing the simultaneous capture of individuals from a single den with genomic estimates of relatedness, enabling the composition of denning groups to be described directly. Several of our records are consistent with these earlier accounts: a mother and daughter sharing a den and three unrelated male–female pairs. Others describe associations not previously reported, including co‐denning between parents and offspring of the opposite sex, full‐siblings and male–female pairs of second‐degree or more distant relationship. Mother–offspring den sharing is thought to be common in greater gliders due to prolonged maternal association during development (Henry 1984; Lindenmayer 2002), but the remaining associations indicate that den sharing is not restricted to breeding pairs and mothers with dependent young.

Two of our records involve more than one male and are less easily reconciled with existing accounts. A nest box contained a family group, comprising a breeding pair together with their adult male offspring (> 1001 g) (Tyndale‐Biscoe and Smith 1969b), and a natural hollow contained two distantly related males with a female unrelated to either. To our knowledge, co‐denning of a breeding pair with their adult offspring has not been previously recorded in this species. Henry (1984) found the ranges of adjacent males to be mutually exclusive and recorded no encounters between males over 41 months, proposing that adult males are intolerant of maturing males. No denning groups comprised males alone in our study, and in both cases the males shared a den in the presence of a female, so the tolerance we observed could be conditional. Nonetheless, the observation of this family group suggests that parental tolerance may extend beyond the juvenile and immature life stages. Such tolerance could facilitate the retention of offspring within or near parental home ranges into adulthood (Waser and Jones 1983) and may contribute to the fine‐scale genetic structure (0–250 m) detected in our study. The lack of prior records may reflect the difficulty of detecting such associations, as confirming which individuals occupy a den simultaneously and how they are related has generally not been possible from observation alone.

Most of the denning groups comprised relatives, and the ecological context of TAL may further inform these den sharing associations. The northern area of the site had recently experienced fire and clear‐felling resulting in the loss of approximately 50 hollow‐bearing trees (Gracanin et al. 2025). In habitats with reduced hollow availability, mountain brushtail possums shift from kin avoidance to kin preference in den sharing (Banks et al. 2011), a behaviour documented following wildfire‐induced loss of hollow‐bearing trees (Banks et al. 2012). Although taxonomically distinct, both southern greater gliders and mountain brushtail possums are obligate hollow users that occur together in eucalypt forests (Lindenmayer et al. 1990), making this comparison informative. The sudden reduction in local hollow availability at TAL may have promoted kin tolerance in den sharing, or perhaps even kin preference (Banks et al. 2011), as a facultative response. Interpretation is complicated by the nest boxes at TAL, which were installed to offset this loss and in which many of the denning groups were recorded. Presumably, co‐denning is more readily detected in nest boxes than natural hollows, and the provision of additional dens may itself promote aggregation independently of any change in social tolerance. However, co‐denning was also recorded in natural hollows, including one triad containing two distantly related males, indicating that these associations are not strictly confined to nest boxes. Whether this reflects typical denning behaviour in southern greater gliders or a facultative response induced by the loss of hollow‐bearing trees remains an important question for further study.

### 4.5 Limitations

The analyses in this study were influenced by the challenges of studying a cryptic, strictly arboreal, endangered species. Sampling constraints affected the robustness of sex‐specific comparisons. Insufficient sample sizes precluded spatial autocorrelation analysis for females at MTL and males at SMB, and limited statistical power for formal tests (e.g., *n* = 4 males at SMB), a common constraint in genetic studies of dispersal (Goudet et al. 2002). Detecting distant dispersal is also inherently difficult, and our sampling was necessarily opportunistic, structured around locating and capturing gliders along transects (MTL; SMB) or from dens (TAL). Long‐distance dispersal events may therefore be underrepresented in our data. These limitations were unavoidable given the difficulty of locating gliders suitable for capture and the inability to reliably distinguish sexes from the ground. We caution that while our data indicate female dispersal at SMB, we do not claim this as evidence of sex‐biased dispersal.

We classified offspring as adults using body mass thresholds (> 1001 g) from Tyndale‐Biscoe and Smith (1969b). While body mass is an imperfect proxy for maturity, several lines of evidence suggest that the adult offspring (1001–1442 g) had reached an age at which dispersal would typically occur (~1 year) (Lindenmayer and Lacy 1995). First, all individuals captured using the branch‐shake method were foraging independently. Second, immature offspring were found > 700 m from parents, indicating that individuals have the physical capacity to disperse longer distances prior to reaching adult body mass, consistent with the findings of Taylor et al. (2007). Third, the consistent proximity of offspring > 1000 g to parents suggests local settlement of adults, rather than transient presence. Nevertheless, we cannot definitively rule out that some individuals classified as adults had not yet dispersed to establish independent home ranges.

### 4.6 Conservation Implications and Future Directions

These findings provide a framework to guide the conservation management of southern greater gliders. The key implications for conservation are that: (i) population genetic health is closely linked to forest connectivity and size, and (ii) limited dispersal is a life history trait that must be considered in conservation management and planning.

Here we have shown that populations in large, connected forests retain higher genomic diversity and a larger effective population size, whereas fully isolated forests exhibit severe genetic erosion and elevated extinction risk. The key conservation priority is therefore to maintain and enhance habitat connectivity through the protection of remnant corridors and active restoration efforts. Modelling corridor pathways suitable for southern greater gliders (Gracanin and Mikac 2023) represents an important preliminary step for management in forests that have experienced or are undergoing fragmentation. Following this, targeted revegetation with preferred *Eucalyptus feed* trees may begin to restore habitat connectivity, and consequently, gene flow (Gracanin and Mikac 2023). Whether habitat restoration can succeed within management‐relevant timescales, and gliders will recolonise and use restored areas as corridors, remains uncertain. Given the uncertainty of restoration success, the preservation of existing eucalypt forest remnants should be prioritised. Such remnants may act as critical ‘stepping stones’ for the dispersal of southern greater gliders (Taylor et al. 2007), although their effectiveness will depend on the hostility of the surrounding matrix.

While maintaining connectivity is the proactive ideal, our findings also provide guidance for populations in a critical state for which intervention is necessary. We provide evidence‐based justification for source selection, should translocations be required to promote healthy, biodiverse populations (Griffith et al. 1989; Weeks et al. 2011). The sociality of southern greater gliders, as revealed through their varied co‐denning associations, must be considered. Given that co‐denning individuals can range from unrelated breeding pairs to entire family groups, we recommend against sourcing single individuals from hollows occupied by multiple gliders. Instead, denning groups should be translocated as intact units, as maintaining familiarity with conspecifics can reduce stress exposure and improve translocation success (Dickens et al. 2010). This approach may also help mitigate fitness costs associated with disrupting established social bonds (Shier and Swaisgood 2012; Goldenberg et al. 2019). While translocating intact social groups helps maximise animal welfare and survival, due to high philopatry and the co‐denning of kin, managers must take care to translocate multiple, unrelated denning groups to ensure that distinct genomic diversity is being introduced.

Incorporating evolutionary and ecological considerations into source population selection is essential (Houde et al. 2015). For translocations, this means identifying source populations that are from environments similar to the release site to ensure they have pre‐existing adaptations for local fitness and also harbour sufficient genetic variation for future adaptive potential (Houde et al. 2015). Due to limited dispersal and genomic similarity up to 250 m, as a general guideline we recommend that denning groups (or individual gliders) be sampled from distances exceeding 250 m to maximise the captured genomic diversity. However, given that patterns of genetic structure at greater distances can vary among populations due to dispersal in some contexts (e.g., positive autocorrelation re‐emerging at 750–1000 m in SMB females), this 250 m guideline should not be treated as universally sufficient to achieve a genomically diverse sample. Beyond genomic sampling design, source individuals should be free of pathogens such as *Chlamydia* (Clough et al. 2024) and have microbiomes well suited to feeding on *Eucalyptus* spp. available at the release site, as recommended for another specialist eucalypt folivore, the koala ( *Phascolarctos cinereus* ) (Blyton et al. 2023). Ultimately, source selection should be appropriately tailored to the destination population to maximise conservation outcomes.

The complete geographic isolation of SMB means gene flow from outside sources is functionally absent, rendering passive conservation insufficient (Gracanin and Mikac 2023). Instead, consideration should be given to management interventions aimed at improving population genetic health and fitness in the short‐term, until connectivity of the Park with contiguous forest ~10 km to the west can be restored (Gracanin and Mikac 2023). The urgency for intervention at SMB is underscored by its critically low *N*_*e*_ (refined to ~35 in this study based on 2024 data) (Frankham et al. 2014) and recent evidence of temporal genetic decline (Clough et al. 2025). As the genomic divergence observed at SMB is recent and drift‐induced, rather than driven by historical differences, this population is an excellent candidate for assisted gene flow and genetic rescue (Frankham 2015; Whiteley et al. 2015). Notably, SMB has been identified as harbouring putative adaptive genetic variation associated with temperature (Knipler et al. 2023), which may be important for persistence under a warming climate. Rather than precluding intervention, this reinforces the need for careful source population selection so that beneficial gene flow does not erode locally adaptive variation. A key priority for future research is to formally assess the feasibility of a genetic rescue program for the SMB population. Such a program could restore genetic variation and improve the long‐term viability of this small and isolated, but evolutionarily significant, population.

## 5 Conclusions

Forest fragmentation has profound impacts on the population genomics of the southern greater glider. Large, continuous forests support populations with higher genomic diversity and low relatedness, while geographic isolation drives genomic erosion and elevated relatedness in small, isolated forests. Our findings refine understanding of the species' dispersal ecology: dispersal is typically limited, creating fine‐scale spatial genetic structure in both continuous and isolated forests. While most offspring appear philopatric, remaining in close proximity to their parents, some dispersal was detected in the isolated forest. Furthermore, co‐denning between related and unrelated conspecifics, including a breeding pair with their adult offspring, reveals a sociality beyond maternal and mating associations alone. These findings underscore the conservation priority of maintaining forest connectivity for southern greater gliders and inform genetic management strategies for populations in need of intervention.

## Author Contributions

**Jordyn Clough:** conceptualization (equal), data curation (lead), formal analysis (lead), funding acquisition (lead), investigation (lead), methodology (equal), project administration (equal), writing – original draft (lead), writing – review and editing (equal). **Ana Gracanin:** conceptualization (supporting), investigation (supporting), resources (supporting), supervision (supporting), writing – review and editing (equal). **Katarina M. Mikac:** conceptualization (equal), funding acquisition (supporting), investigation (supporting), methodology (equal), project administration (equal), resources (lead), supervision (lead), writing – review and editing (equal).

## Funding

This work was supported by Holsworth Wildlife Research Endowment—Equity Trustees Charitable Foundation and the Ecological Society of Australia.

## Conflicts of Interest

The authors declare no conflicts of interest.

## Supporting information


**Appendix S1:** Supporting results.

## Data Availability

The dataset generated during the current study is available from the Figshare digital repository: https://doi.org/10.6084/m9.figshare.31937535.

## Appendix A

**TABLE A1:** Site characteristics for the three sampled southern greater glider populations.

Site	Elevation	Forest type	Burn status
MTL	587 m–673 m	Dry sclerophyll forest	Unburnt
TAL	893 m–1096 m	Wet sclerophyll forest/dry sclerophyll forest	Burnt (2019/20)
SMB	9 m–26 m	Dry sclerophyll forest	Unburnt

*Note:* Elevation range, forest type and burn status during the 2019/20 Australian bushfire season are indicated for Mount Lindsey (MTL), Tallaganda (TAL) and Seven Mile Beach National Park (SMB). Forest types follow the vegetation formations defined in Keith (2004).

**TABLE A2:** Pairwise beta diversity of southern greater glider populations at Mount Lindsey (MTL), Tallaganda (TAL) and Seven Mile Beach National Park (SMB).

Population comparison	^*0*^ *D*_*β*_	^*2*^ *D*_*β*_
MTL–TAL	1.17 (± 0.238)	1.02 (± 0.124)
MTL–SMB	1.19 (± 0.243)	1.05 (± 0.180)
TAL–SMB	1.20 (± 0.246)	1.07 (± 0.124)

*Note:* Analysis based on 6792 SNPs. Values show mean (± SD).
