# Supplementary material for: Limited Dispersal and Fragmentation‐Induced Genomic Erosion in Southern Greater Gliders (Petauroides volans)
Source: Ecol Evol. 2026 Aug 16;16(8):e74204. doi: 10.1002/ece3.74204 (PMC13477829; doi:10.1002/ece3.74204)
Supplement: Supplementary file 1 [file ECE3-16-e74204-s001.docx]

**Limited Dispersal and Fragmentation‐Induced Genomic Erosion in Southern Greater Gliders (*Petauroides volans*)**

Jordyn Clough^1*^, Ana Gracanin^2^, Katarina Mikac^1^

^1^Environmental Futures, Faculty of Science, Medicine and Health, School of Science, University of Wollongong, Wollongong, New South Wales, Australia

^2^Fenner School of Environment and Society, Australian National University, Canberra, Australian Capital Territory, Australia

^*^Correspondence

**Appendix S1: Supporting results**

**Text S1.** Genomic identity by descent (IBD) analysis of TAL dyad #110–#80

Coancestry (*θ* = 0.248) of #110–#80 is consistent with a first-degree genomic relationship (*θ* ≈ 0.25). Parent–offspring (PO) are expected to be IBD1 (share exactly one allele) at all loci (*Δ_8_* ≈ 1). Dyad fails to share exactly one allele at 57% of loci (*Δ_8_* = 0.430) therefore PO excluded. Half-siblings (HS) are expected to be IBD0 (share zero alleles) at ~50% of loci (*Δ_9_* ≈ 0.5). Dyad is IBD0 at only 9% of loci (*Δ_9_* = 0.094), therefore HS excluded. Full-siblings (FS) are expected to be IBD2 (share two alleles) at ~25% of loci (*Δ_7_* ≈ 0.25). Dyad is IBD2 at 6% of loci (*Δ_7_* = 0.057), but substantial probability exists in inbred identity states (*Δ_1_*… *Δ_6_*). Total magnitude of two-allele sharing (*Δ_7_* + ∑(*Δ_1_*… *Δ_6_*) = 0.476) is elevated beyond the theoretical expectation of ~25% for non-inbred FS. This is consistent with FS from highly related parents (inbred FS). The observed variance in inbreeding coefficients for #110 (*F* = 0.295) and #80 (*F* = 0.153) reflects the stochastic nature of Mendelian segregation and recombination in an inbred lineage, where individuals can inherit disparate proportions of the parental autozygome.
